# Weekly pattern of alcohol-attributable male mortality before and after imposing limits on hours of alcohol sale in Lithuania in 2018

**DOI:** 10.1177/14034948231184288

**Published:** 2023-07-04

**Authors:** Daumantas Stumbrys, Mindaugas Štelemėkas, Domantas Jasilionis, Jürgen Rehm

**Affiliations:** 1 Health Research Institute, Faculty of Public Health, Lithuanian University of Health Sciences, Lithuania; 2 Institute of Sociology, Lithuanian Centre for Social Sciences, Lithuania; 3 Demographic Research Centre, Vytautas Magnus University, Lithuania; 4 Demographic Data Laboratory, Max Planck Institute for Demographic Research, Germany; 5 Institute for Mental Health Policy Research and Campbell Family Mental Health Research Institute, Centre for Addiction and Mental Health (CAMH), Canada; 6 Dalla Lana School of Public Health, Institute of Health Policy, Management and Evaluation, and Department of Psychiatry, University of Toronto (UofT), Canada; 7 PAHO/WHO Collaborating Centre at CAMH, Canada; 8 WHO European Region Collaborating Centre at Public Health Institute of Catalonia, Spain; 9 Technische Universität Dresden, Klinische Psychologie and Psychotherapie, Germany; 10 Zentrum für Interdisziplinäre Suchtforschung der Universität Hamburg (ZIS), Universitätsklinikum Hamburg-Eppendorf, Hamburg, Germany

**Keywords:** Alcohol-attributable mortality, alcohol poisoning, alcohol control policy, weekly pattern of deaths, Lithuania

## Abstract

**Aims:**

From 1 January 2018, the number of retail hours for the sale of alcohol was reduced from 14 to 5 hours on Sundays and from 14 to 10 hours on the other days of the week in Lithuania. The significant reduction of hours for the sale of alcohol on Sundays may have affected the distribution of alcohol-attributable deaths during the week. This study aimed to examine the change in the weekly pattern of alcohol-attributable male mortality before and after imposing limits on the hours when alcohol can be sold.

**Methods:**

Age-standardised male death rates by days of the week were calculated for four groups according to cause of death: alcohol poisoning (X45), all external causes of death (V01–Y98), diseases of the circulatory system (I00–I99) and all other causes of death. We compared age-standardised death rates for two periods: before (2015–2017) and after (2018–2019) the intervention. Mortality and population data were obtained from the Lithuanian Institute of Hygiene and Human Mortality Database.

**Results:**

We found that during 2018–2019, earlier observed peak in age-standardised death rates for external causes of death on Sunday diminished, and this day no longer differed from the weekly average. The same tendency was also observed for the Monday excess mortality due to circulatory diseases.

**Conclusions:**

**The reduction of the hours when alcohol can be sold from the beginning of 2018 was associated with a change in a weekly pattern of alcohol-attributable male mortality. However, more studies are needed to examine the causes of the change in mortality pattern.**

## Introduction

Previous epidemiological studies have found increased alcohol-attributable mortality at the weekend and on Monday in Lithuania and other post-Soviet countries [1–3]. Accordingly, a study conducted in Moscow found a significant increase in deaths from alcohol poisoning, injury, violence and cardiovascular diseases on Saturdays, Sundays and Mondays between 1993 and 1995 [2]. Such a mortality excess is related to an extremely harmful drinking pattern which is characterised as a period of continuous drunkenness lasting several days. This phenomenon is also called *zapoi* [4]. Similar alcohol-attributable mortality patterns were found in Lithuania between 1988 and 1997 [1]. Furthermore, Radisauskas et al. [3] found significantly higher post-mortem blood or urine alcohol levels during the weekend among out-of-hospital deaths (age 25–64 years) from ischaemic heart disease in Kaunas (Lithuania) between 1993 and 2007. Binge drinking has been hypothesised as the dimension of drinking causing this excess mortality, as it has been causally linked to all forms of injury, including intentional injury [5], and serves as a catalyst for pathophysiological cardiovascular events by increasing blood pressure, cardiac rhythm and coagulability [1].

Between 2008 and 2009, several evidence-based alcohol control policies (e.g. increased excise taxation, ban on off-premises alcohol sales, restricted marketing during the daytime and measures to prevent drink driving) came into effect in Lithuania which may have contributed to reversing negative alcohol-attributable harm indicators such as mortality [6,7]. However, the assessment of the impact of alcohol control policies in that period may have been complicated by the effects of the global economic crisis of 2008–2010 which may have contributed due to the affordability of alcohol being reduced [8,9].

Another period with increased alcohol control policies is between 2014 and 2020 when Lithuania introduced several comprehensive alcohol control measures [8,10]. In 2014–2015, excise taxation was increased. In 2016, alcohol sales were banned at petrol stations, and excise tax was increased. Drink driving became a criminal offence, and the alcohol excise tax was increased multiple times, including a major increase in 2017. Furthermore, the legal minimum age increased from 18 to 20 years; TV, radio and Internet advertising was banned; and off-premises sales hours were reduced substantially in 2018. In 2019, the alcohol excise tax for spirits was increased by 10%, and selling food, toys and other goods with designs mimicking alcoholic beverages were banned. Finally, sales from non-stationary points of sale were banned, temporary alcohol sale licenses were revoked and alcohol tax for spirits increased again in 2020 by 10% [6,8,10].

In addition, the retail hours for off-premises alcohol sales were reduced to 10:00–20:00 from Monday to Saturday and to 10:00–15:00 on Sunday since 1 January 2018. The number of retail hours for alcohol sales was reduced from 14 to 5 hours on Sundays and from 14 to 10 hours on other days of the week from the beginning of 2018 [11]. While all of these policies should have some effects on the level of consumption and attributable harm by pushing down the annual mortality trend, the availability restrictions arguably can be hypothesised specifically to have had the largest effect on binge drinking and attributable harm such as injury and violence [12]. This restriction may be considered to have had a specific effect on the weekly mortality pattern (especially for Sundays and Mondays), despite other policy measures introduced before, after or together with the reduction of hours when alcohol can be sold. According to the availability theory [13], the reduction in hours when alcohol can be sold should lead to an overall reduction in mortality, and the largest decrease in hours on Sundays should furthermore have an effect on the distribution of alcohol-attributable deaths during the week.

The goal of this study was to examine the change in weekly pattern of alcohol-attributable deaths among males aged 20–79 years before and after the limits on hours for the sale of alcohol were imposed in Lithuania in 2018. We aimed to explore: (a) whether the weekly pattern of alcohol-attributable mortality before the intervention reflected patterns of alcohol-attributable mortality captured in previous studies, (b) the overall change in alcohol-attributable mortality in different groups according to cause of death after the intervention and (c) the change in the weekly pattern of alcohol-attributable mortality in different groups according to cause of death.

## Methods

### Data sources and data characteristics

Mortality and population data were obtained from the Lithuanian Institute of Hygiene and Human Mortality Database [14]. Prior studies suggest that cause-specific mortality data for Lithuania is reliable and highly complete [15]. The number of deaths and person years were divided into five-year age groups. We examined two periods: before (2015–2017) and after (2018–2019) the limits on hours for the sale of alcohol were imposed in Lithuania in 2018. Both periods represent a stable period of moderate growth for the Lithuanian economy until the beginning of the COVID-19 pandemic in 2020. Males aged 20–79 years were selected as the target group. Previous studies have shown that this population group experience the highest alcohol-attributable mortality rates in Lithuania [7].

### Measures and statistical analysis

Age-standardised death rates (SDR) per 100,000 people for different groups according to cause of death were calculated for each day of the week. The European standard population for 2013 [16] was applied in calculations. Cause of death was classified according to the International Statistical Classification of Diseases and Related Health Problems, 10th edition. Four groups for cause of death were analysed in our research, three of which were directly or indirectly attributable to alcohol: (a) alcohol poisoning (X45), (b) external causes of death (V01–Y98) except alcohol poisoning (X45); (c) diseases of the circulatory system (I00–I99) and (d) all other causes of death. Data were analysed using IBM SPSS Statistics for Windows v20.0 (IBM Corp., Armonk, NY) and MS Excel. We calculated 95% confidence intervals for SDR values for each day of the week and performed an additional test: a one-sample *t-*test to test for the difference between the SDR values for each day of the week and the weekly average.

### Ethics

The authors of this study analysed individual-level anonymous data. Ethics approval for this study was not applicable in accordance with the regulations of academic ethics procedures in the Republic of Lithuania.

## Results

We found that the number of deaths and SDRs decreased in all three cause-of-death groups (alcohol poisoning, external causes of death and diseases of the circulatory system) during the reference period (see Tables I and II). Furthermore, all changes in the weekly average SDRs were statistically significant. Results showed that weekly average SDRs for alcohol poisoning decreased from 2.35 to 1.75 (*p*<0.001), SDRs for external causes of death decreased from 28.09 to 22.71 (*p*=0.001), and SDRs for circulatory diseases decreased from 102.99 to 91.75 (*p*<0.001). However, the change in the weekly average SDR for the remaining causes of death was not statistically significant. The weekly average SDR for all other causes of death showed a modest decrease from 107.46 to 104.49 (*p*=0.097).

**Table I.** The total number of male deaths (20–79 years old) in Lithuania before and after the intervention in 2018.

Day of the week	Alcohol poisoning				External causes				Circulatory disease				All other causes			
	2015–2017		2018–2019		2015–2017		2018–2019		2015–2017		2018–2019		2015–2017		2018–2019	
	*n*	%	*n*	%	*n*	%	*n*	%	*n*	%	*n*	%	*n*	%	*n*	%
Monday	77	15.9	32	13.2	841	15.0	430	14.3	2637	15.1	1531	14.8	2736	14.4	1851	15.1
Tuesday	66	13.6	39	16.1	760	13.5	438	14.6	2510	14.3	1593	15.4	2775	14.6	1776	14.5
Wednesday	68	14.0	29	12.0	710	12.6	413	13.8	2492	14.2	1352	13.1	2752	14.5	1781	14.5
Thursday	58	12.0	32	13.2	727	12.9	375	12.5	2403	13.7	1437	13.9	2729	14.4	1769	14.4
Friday	65	13.4	32	13.2	758	13.5	433	14.4	2461	14.1	1441	13.9	2657	14.0	1679	13.7
Saturday	75	15.5	35	14.5	862	15.3	481	16.0	2524	14.4	1469	14.2	2694	14.2	1651	13.4
Sunday	76	15.7	43	17.8	959	17.1	428	14.3	2478	14.2	1519	14.7	2648	13.9	1773	14.4
Overall	485	100	242	100	5617	100	2998	100	17,505	100	10342	100	18,991	100	12,280	100

Data source: Institute of Hygiene (Lithuania).

**Table II.** The SDRs and their 95% confidence intervals (95% CI) for males (20–79 years old) for four cause-of-death groups in Lithuania before (2015–2017) and after (2018–2019) the intervention on 1 January 2018.

Day of the week	2015-2017		2018-2019	
	SDR (95% CI)	*p*-Value	SDR (95% CI)	*p*-Value
*Alcohol poisoning*				
Monday	**2.63** (2.04, 3.21)	0.021	1.63 (1.07, 2.20)	0.230
Tuesday	2.22 (1.68, 2.75)	0.176	1.98 (1.36, 2.60)	0.048
Wednesday	2.31 (1.76, 2.86)	0.678	1.44 (0.91, 1.96)	0.012
Thursday	1.99 (1.48, 2.51)	0.007	1.62 (1.06, 2.18)	0.178
Friday	2.21 (1.67, 2.74)	0.147	1.67 (1.09, 2.24)	0.367
Saturday	2.55 (1.97, 3.12)	0.073	1.81 (1.21, 2.41)	0.534
Sunday	2.57 (1.99, 3.14)	0.052	**2.14** (1.50, 2.78)	0.005
Average	2.35 (1.80, 2.91)		1.75 (1.17, 2.34)	
*External causes*				
Monday	29.88 (27.86, 31.90)	0.164	23.02 (20.84, 25.20)	0.633
Tuesday	26.60 (24.71, 28.49)	0.238	23.25 (21.08, 25.43)	0.409
Wednesday	25.16 (23.31, 27.01)	0.042	21.94 (19.82, 24.05)	0.250
Thursday	25.43 (23.58, 27.28)	0.058	19.99 (17.96, 22.01)	0.004
Friday	26.60 (24.71, 28.50)	0.239	22.90 (20.74, 25.06)	0.767
Saturday	29.48 (27.51, 31.44)	0.266	**25.39** (23.12, 27.66)	0.005
Sunday	**33.46** (31.34, 35.57)	0.003	22.50 (20.37, 24.63)	0.740
Average	28.09 (26.14, 30.03)		22.71 (20.56, 24.86)	
*Circulatory diseases*				
Monday	**107.98** (103.86, 112.10)	0.003	94.31 (89.59, 99.04)	0.202
Tuesday	103.61 (99.56, 107.66)	0.564	**99.31** (94.43, 104.18)	0.006
Wednesday	102.81 (98.77, 106.84)	0.857	84.26 (79.77, 88.75)	0.006
Thursday	99.32 (95.35, 103.29)	0.011	89.31 (84.69, 93.93)	0.221
Friday	101.25 (97.25, 105.25)	0.136	89.83 (85.19, 94.47)	0.324
Saturday	103.73 (99.69, 107.78)	0.493	91.34 (86.67, 96.01)	0.826
Sunday	102.26 (98.23, 106.29)	0.495	93.90 (89.18, 98.62)	0.275
Average	102.99 (98.96, 107.03)		91.75 (87.07, 96.43)	
*All other causes of death*				
Monday	108.46 (104.40, 112.53)	0.223	**110.58** (105.54, 115.61)	0.006
Tuesday	**109.85** (105.76, 113.94)	0.018	106.26 (101.32, 111.21)	0.276
Wednesday	**109.45** (105.36, 113.54)	0.035	105.79 (100.87, 110.70)	0.416
Thursday	107.57 (103.53, 111.60)	0.893	105.06 (100.16, 109.95)	0.717
Friday	105.41 (101.40, 109.42)	0.031	**100.16** (95.37, 104.95)	0.026
Saturday	106.75 (102.72, 110.78)	0.368	98.96 (94.19, 103.74)	0.010
Sunday	104.75 (100.76, 108.74)	0.010	104.66 (99.79, 109.53)	0.915
Average	107.46 (103.42, 111.51)		104.49 (99.60, 109.38)	

*p*-Values denote differences from the weekly average. SDRs for a specific day of the week that are statistically higher (*p*<0.05) than the average SDR are shown in bold. *p*-Values refer to one-sample *t-*tests examining whether the average SDRs are statistically different from SDRs for each specific day of the week. Data source: Institute of Hygiene (Lithuania).

Table II shows SDRs and their 95% confidence intervals by days of the week for different cause-of-death groups for males in Lithuania. *p-*Values for the one-sample *t*-test examining whether the average SDR is statistically different from each specific day of the week SDR are also presented in Table II. Our analysis showed that the SDR for alcohol poisoning was higher on Monday (SDR=2.63, *p*=0.021), Saturday (SDR=2.55, *p*=0.073) and Sunday (SDR=2.57, *p*=0.052) compared to the weekly average (SDR=2.35) between 2015 and 2017. However, the difference was not statistically significant for Saturday and Sunday. The pattern of the SDRs for alcohol poisoning changed slightly between 2018 and 2019 (see Table II). During that period, the SDR for alcohol poisoning on Monday (SDR=1.63, *p*=0.230) decreased below the weekly average (SDR=1.75). However, it remained above the weekly average on Sunday (SDR=2.14, *p*=0.005).

Between 2015 and 2017, the SDR for external causes of death was higher on Monday (SDR=29.88, *p*=0.164), Saturday (SDR=29.48, *p*=0.266) and Sunday (SDR=33.46, *p*=0.003) compared to the weekly average (SDR=28.09). Furthermore, on Sunday, the difference was statistically significant from the weekly average SDR. We found that after 1 January 2018, the SDR for external causes of death on Sunday (SDR=22.50, *p*=0.740) did not statistically differ from the weekly average (SDR=22.71).

Between 2015 and 2017, SDR for circulatory diseases was statistically significantly higher on Monday (107.98, *p*=0.003) compared to the weekly average (SDR=102.99). We found that between 2018 and 2019, the excess mortality for circulatory diseases on Monday (SDR=94.31, *p*=0.202) decreased and was not statistically significant from the weekly average (SDR=91.75). Furthermore, the SDR for circulatory disease became statistically significantly higher on Tuesday (99.31, *p*=0.003) compared to the weekly average in 2018–2019.

## Discussion

In this study, we explored the weekly pattern of alcohol-attributable male mortality. We tested whether alcohol-attributable mortality by day of the week reflected patterns of alcohol-attributable mortality captured in previous studies in Lithuania. Furthermore, we explored whether the pattern of weekly pattern of alcohol-attributable mortality changed after imposing limits on the hours when alcohol could be sold in Lithuania in 2018. Understanding the associations between the reduction in hours for the sale of alcohol and changes in excess mortality at the weekend and on Monday is important for planning alcohol policy interventions.

The 2014–2020 time period is marked with several important alcohol control policies targeting the whole population or smaller subgroups [17]. Only one of the implemented measures targeted the hours when alcohol could be sold both in general as well as on a specific day (Sunday), and this was the focus of this study. When the off-premises hours for the sale of alcohol in Lithuania were restricted on Sundays more than on any other day of the week on 1 January 2018, the main argument for policymakers was that people tend to drink more during the weekends, resulting in a proportion of the population not becoming sober until the beginning of the next week. The decision may have been supported by arguments from the past studies [3] as well as by data from preliminary alcohol brief testing results from workplaces [18]. This analysis has provided additional arguments to support the implemented policy for more restrictive retail sale hours for alcoholic beverages on Sundays by showing that higher alcohol intoxication incidence rates were found on Mondays and in the morning than on any other day or at any other time [18].

The alternative analysis also included the year 2020. The year 2020 was the first year of the COVID-19 pandemic marked by a very strict countrywide lockdown from 16 March to 16 June. This lockdown resulted in the closure of many public places, including bars and restaurants during the initial stage of the pandemic, and the restrictions were then gradually lifted. Another national quarantine period was introduced in November 2020 [19]. Therefore, we decided not to include 2020 in the main analysis because of notable increases and sudden changes in overall weekly mortality during this period. However, Lithuania did not close traditional outlets where off-premises alcohol sales were available, nor did it introduce any additional alcohol control policies targeting the availability of off-premises alcohol sales. Even though the pandemic has resulted in some behavioural changes to drinking alcohol for some people, a population survey data from 2020 indicated that there were no major behavioural shifts regarding drinking alcohol among most of the Lithuanian population [20,21] that would warrant disregarding the year 2020 from the analysis. Moreover, Lithuania was one of the few countries in Europe which slightly increased its level of alcohol consumption during 2020 [22,23]. The results of the alternative analysis showed that the COVID-19 pandemic was not a game changer in the weekly pattern of alcohol-attributed mortality after 2018. The results of alternative analysis (2018–2020) are provided in the Supplemental Material (see Supplemental Table SI).

There are several limitations to our study that are worth mentioning. First, we found statistically significant changes in the weekly pattern of male mortality for all external causes of death, circulatory diseases and alcohol poisoning. Previous studies based on autopsy data have shown that a large share of external causes of death are attributable to alcohol use in Lithuania [24,25]. Relatively small numbers of deaths due to more specific causes such as alcohol poisoning did not allow to obtain statistically robust results.

Second, in 2018–2019, we found an unexpectedly high SDR for circulatory diseases and alcohol poisoning on Tuesday compared to the weekly average. We believe that the higher mortality on Tuesdays may be related to the relatively high number of Tuesdays that followed public holidays such as New Year’s Eve in 2018–2019. A previous study conducted in Lithuania showed that alcohol intoxication incidence among workers was higher after holidays [18].

Despite these limitations, our study was able to demonstrate that the alcohol control policy of availability restrictions, in particular stricter restrictions of hours when alcohol could be sold on Sundays, may be associated with lower mortality for some causes of male death. We suggest that these policies contributed to the reduction of variability in mortality due to external causes and circulatory diseases between the weekend and weekdays. Both reductions in the levels and variability in these cause-of-death groups among males may be explained by their relationships to prevailing binge drinking patterns. Thus, the availability restrictions should be considered as one of the effective means of reducing public health harm related to binge drinking and its consequences, including mortality.

Unfortunately, the most recent evidence warns about new trends and forms in the alcohol trade contributing to increasing access to alcohol via expanding online delivery services (partly inspired by the COVID-19 restrictions) [26,27]. The policy of reduced off-premises hours for alcohol sales is under constant pressure from interest groups at the Lithuanian Parliament, and there are attempts to revoke the restriction on Sundays [28]. From a public health point of view, such policies need to be examined to provide unbiased evidence to support national and international policymaking processes to safeguard the health of the population.

## Conclusions

This study shows the potential of alcohol control policies to reduce binge drinking–related excess mortality during weekends and Mondays. The evidence from Lithuania showed that imposing limits on the hours when alcohol can be sold in Lithuania in 2018 was followed by a change in the weekly pattern of alcohol-attributable male mortality. Specifically, there was a significant decrease in excess mortality for circulatory diseases on Monday and a significant decrease in excess mortality for external causes of death on Sunday. However, more studies are needed to examine the causes of the change in mortality pattern.

## Supplemental Material

sj-docx-1-sjp-10.1177_14034948231184288 – Supplemental material for Weekly pattern of alcohol-attributable male mortality before and after imposing limits on hours of alcohol sale in Lithuania in 2018 Supplemental material, sj-docx-1-sjp-10.1177_14034948231184288 for Weekly pattern of alcohol-attributable male mortality before and after imposing limits on hours of alcohol sale in Lithuania in 2018 by Daumantas Stumbrys, Mindaugas Štelemėkas, Domantas Jasilionis and Jürgen Rehm in Scandinavian Journal of Public Health
