# Supplementary material for: Weekly pattern of alcohol-attributable male mortality before and after imposing limits on hours of alcohol sale in Lithuania in 2018
Source: Scand J Public Health. 2023 Jul 4;52(6):698–703. doi: 10.1177/14034948231184288 (PMC10877377; doi:10.1177/14034948231184288)
Supplement: sj-docx-1-sjp-10.1177_14034948231184288 – Supplemental material for Weekly pattern of alcohol-attributable male mortality before and after imposing limits on hours of alcohol sale in Lithuania in 2018 [file sj-docx-1-sjp-10.1177_14034948231184288.docx]

**Table S1.** The SDRs and their 95% confidence intervals (CI 95%) for males (20-79 years old) for four groups of causes of death in Lithuania after the intervention on the 1^st^ January 2018 (2018-2020, **including the 2020 pandemic year**).

| Day of the week | Alcohol poisoning | | External causes | |
| --- | --- | --- | --- | --- |
|  | **SDR (CI 95%)** | ***p*-value** | **SDR (CI 95%)** | ***p*-value** |
| Monday | 1.82 (1.33, 2.30) | 0.136 | 22.18 (20.44, 23.92) | 0.527 |
| Tuesday | 1.61 (1.15, 2.07) | 0.627 | 22.87 (21.11, 24.63) | 0.429 |
| Wednesday | 1.30 (0.90, 1.71) | 0.008 | 23.05 (21.28, 24.82) | 0.260 |
| Thursday | 1.53 (1.09, 1.97) | 0.204 | 20.23 (18.57, 21.90) | 0.003 |
| Friday | 1.50 (1.06, 1.94) | 0.130 | 22.02 (20.29, 23.75) | 0.345 |
| Saturday | 1.86 (1.37, 2.35) | 0.068 | **24.13** (22.32, 25.93) | 0.011 |
| Sunday | **1.99** (1.48, 2.50) | 0.011 | 22.91 (21.15, 24.66) | 0.387 |
| Average | 1.66 (1.20, 2.12) |  | 22.48 (20.74, 24.23) |  |

*P*-values denote differences from the weekly average.

| Day of the week | Circulatory diseases | | All other causes | |
| --- | --- | --- | --- | --- |
|  | **SDR (CI 95%)** | ***p*-value** | **SDR (CI 95%)** | ***p*-value** |
| Monday | 97.43 (93.51, 101.35) | 0.113 | **114.28** (110.11, 118.45) | 0.031 |
| Tuesday | **99.33** (95.36, 103.30) | 0.009 | 113.54 (109.38, 117.70) | 0.074 |
| Wednesday | 92.87 (89.02, 96.72) | 0.029 | 112.07 (107.94, 116.19) | 0.413 |
| Thursday | 96.26 (92.35, 100.18) | 0.540 | 113.24 (109.09, 117.39) | 0.107 |
| Friday | 93.61 (89.76, 97.46) | 0.082 | 107.35 (103.29, 111.40) | 0.018 |
| Saturday | 92.78 (88.94, 96.62) | 0.026 | 106.76 (102.73, 110.80) | 0.010 |
| Sunday | 97.15 (93.23, 101.07) | 0.169 | 110.17 (106.09, 114.26) | 0.471 |
| Average | 95.63 (91.74, 99.53) |  | 111.06 (106.95, 115.17) |  |

*P*-values denote differences from the weekly average.

Notes. Marked in **bold**: SDR for a specific day of the week is statistically higher than the average SDR (*p*<0.05). *P-*values refer to one-sample *t*-test examining whether the average SDRs are statistically different from SDRs for each specific day of the week. Data source: Institute of Hygiene (Lithuania).
